# The application of Global Trigger Tool in monitoring antineoplastic adverse drug events: a retrospective study

**DOI:** 10.3389/fonc.2024.1230514

**Published:** 2024-05-08

**Authors:** Yang Liu, Xianjun Liu, Binbin Xia, Jing Chen, Wenfang Sun, Fang Liu, Hua Cheng

**Affiliations:** Department of Pharmacy, Beijing Luhe Hospital, Capital Medical University, Beijing, China

**Keywords:** adverse drug reactions/events, Global Trigger Tool (GTT), antineoplastic drugs, patient safety, risk factors

## Abstract

**Objective:**

This study aimed to establish an antineoplastic drugs trigger tool based on Global Trigger Tool (GTT), to examine the performance by detecting adverse drug events (ADEs) in patients with cancer in a Chinese hospital (a retrospective review), and to investigate the factors associating with the occurrence of antineoplastic ADEs.

**Methods:**

Based on the triggers recommended by the GTT and those used in domestic and foreign studies and taking into account the scope of biochemical indexes in our hospital, some of them were adjusted. A total of 37 triggers were finally developed. Five hundred medical records of oncology patients discharged in our hospital from 1 June 2020 to 31 May 2021 were randomly selected according to the inclusion and exclusion criteria. These records were reviewed retrospectively by antineoplastic drugs trigger tool. The sensitivity and specificity of the triggers were analyzed, as well as the characteristics and risk factors for the occurrence of ADEs.

**Results:**

Thirty-three of the 37 triggers had positive trigger, and the sensitivity rate was 91.8% (459/500). For the specificity, the positive predictive value of overall ADEs was 46.0% (715/1556), the detection rate of ADEs was 63.0% (315/500), the rate of ADEs per 100 admissions was 136.0 (95% CI, 124.1–147.9), and the rate of ADEs per 1,000 patient days was 208.33 (95% CI, 201.2–215.5). The top three antineoplastic drugs related to ADEs were antimetabolic drugs (29.1%), plant sources and derivatives (27.1%), and metal platinum drugs (26.3%). The hematologic system was most frequently involved (507 cases, 74.6%), followed by gastrointestinal system (89 cases, 13.1%). Multivariate logistic regression analysis showed that the number of combined drugs (OR = 1.14; 95% CI, 1.07–1.22; *P* < 0.001) and the previous history of adverse drug reaction (ADR) (OR = 0.38; 95% CI, 0.23–0.60; *P* < 0.001) were the risk factors for ADEs. The length of hospital stay (OR = 0.40; 95% CI, 0.14–1.12; *P* < 0.05) and the previous history of ADR (OR = 2.18; 95% CI, 1.07–4.45; *P* < 0.05) were the risk factors for serious adverse drug events (SAE).

**Conclusion:**

The established trigger tool could be used to monitor antineoplastic drugs adverse events in patients with tumor effectively but still needs to be optimized. This study may provide some references for further research in order to improve the rationality and safety of antineoplastic medications.

## 1 Introduction

Adverse drug reactions/events (ADRs/ADEs) are the primary drug safety concern that not only pose a serious threat to public health and increase morbidity and mortality but also impose a heavy economic burden on individuals or countries (1). Malignant tumors have become the second leading cause of death after cardiovascular disease (2). ADEs occur frequently in oncology and justify continuous assessment and monitoring. The annual report of China’s national ADR monitoring in 2021 shows that, among the chemical medicines involved in ADR/ADE reports, antineoplastic drugs rank second; among the chemical medicines involved in the serious ADR/ADE reports, antineoplastic drugs rank first (3). As new treatments and protocols are rapidly introduced, new safety hazards evolve, such as antitumor targeted drug adverse reactions. Immune checkpoint inhibitors (ICIs), such as Programmed cell death receptor 1 (PD-1)/Programmed cell death ligand 1 (PD-L1) monoclonal antibody, are the most recent breakthrough in the treatment of cancer. However, by activating the immune system, ICIs can also lead to excessive immune reactions against healthy normal organs, known as immune-related adverse events (irAEs) (4, 5).

The Institute for Healthcare Improvement (IHI) is a not-for-profit organization, leading the improvement of healthcare throughout the world. IHI helps accelerate change by cultivating promising concepts for improving patient care and turning those ideas into action. Thousands of healthcare providers participate in IHI’s groundbreaking work. The “IHI Global Trigger Tool (GTT) for measuring adverse events” is a new active monitoring method for adverse events, which was developed by the IHI in 2003 (6) and revised in 2009, known as the “Version 2 White Paper.” The IHI GTT for measuring adverse events provides an easy-to-use method for accurately identifying adverse events and measuring the rate of adverse events over time. The Trigger Tool methodology is a retrospective review of a random sample of inpatient hospital records using “triggers” (or clues) to identify possible adverse events. Many hospitals have used this tool to identify adverse events and to assess the level of harm from each adverse event. The use of “triggers” to identify ADEs is an effective method for measuring the overall level of harm from medications in a healthcare organization. The white paper recommends that the triggers listed in the IHI GTT are recommendations only, and organizations are encouraged to modify them based on their own characteristics (7).

## 2 Materials and methods

In recent 10 years, several studies in domestic and overseas had shown the effectiveness of GTT in detecting ADEs in specific groups of people (8–19). In China, the National Center for ADR Monitoring has established the National ADR Monitoring System, a spontaneous reporting system to report ADR/ADE, and has developed standardized grading criteria based on the World Health Organization standards to improve the data quality management of ADR/ADE reporting. However, this is a passive monitoring method; the study found that about 90% of ADEs were underreported, which can delay the detection of safety signals (20). Thus, despite the availability of this surveillance strategy, the incidence and characteristics of ADEs in Chinese patients are largely unknown, especially for specific groups of people or medications (21). To our knowledge, there are only a few localized trigger tools for detecting ADE (8–13), which are more efficient compared to the most widely used Voluntary Reporting Notification in China, and it is also more labor-efficient. This study aim to establish a antineoplastic drugs trigger tool based on GTT, to examine the performance by detecting ADEs in patients with cancer in a Chinese hospital (a retrospective review), and to investigate the factors associating with the occurrence of antineoplastic ADEs, which could provide a reference for the further modification of the trigger tool and more suitable for the local cancer inpatients. In the future, the trigger tool could be incorporated into routine screening systems to provide real-time identification of ADEs, thereby enabling initiation of timely clinical interventions.

### 2.1 Study design

#### 2.1.1 Research subjects

This review was conducted in Beijing Luhe Hospital, Capital Medical University, a tertiary teaching hospital with 1,300 beds. The oncology department has 70 beds and focuses on the standardized diagnosis and drug treatment of malignant tumors such as digestive system tumors (colorectal cancer, esophageal cancer, pancreatic cancer, gastric cancer, and hepatobiliary cancer), lung cancer, breast cancer, gynecological tumors, urogenital system tumors, and neuroendocrine tumors. The hematology department has 45 beds and focuses on the malignant blood diseases: acute leukemia, chronic leukemia, lymphoma, multiple myeloma, etc. A total of 5,839 medical records of discharge from oncology department and hematology department (lymphomas) of our hospital from 1 June 2020 to 31 May 2021 were collected. From the medical records meeting the screening conditions, 500 medical records were randomly selected by Microsoft Excel 2010 software random sampling tool, and the achieved medical records were extracted according to the extracted medical record number for retrospective medical records review. The PASS software was used to calculate the sample size, by single-sample sensitivity and specificity test. Referring to previous literature reports (15), the sensitivity and specificity of the GTT in patients with tumor receiving antitumor drugs were estimated to be 0.8 and 0.8, respectively. The one-sided test was selected: α = 0.025 and 1 – β = 0.90. The final required sample size was 93, including at least 28 cases (take the maximum value). This study is expected to randomly select 500 cases from the medical records that meet the inclusion and exclusion criteria. The incidence of adverse reactions is about 0.3 (17), and it is expected to include 150 cases, which can meet the sample size requirements.

#### 2.1.2 Inclusion and exclusion criteria

### 2.2 Triggers extraction and revision

The medical records were screened according to the following inclusion criteria: patients diagnosed as malignant tumor (includes solid tumor all cancer types and lymphomas), the length of hospital stay is longer than 2 days and no more than 30 days, and patient age is 18 years or older. Exclusion criteria: no antineoplastic drugs treatment.

This study attempts to establish a trigger tool based on GTT to detect ADEs in patients with cancer, and the trigger tool was established by three steps including literature research, trigger extraction and revision, and expert investigation. In this study, the 13 medication modules recommended in the white paper of GTT and the triggers of patients with tumor in literatures were referred to construct 34 initial triggers (8, 14–19). According to the Common Terminology Criteria for Adverse Events (CTCAE) version 5.0 issued by the National Cancer Institute of the United States (22), combined with the scope of our hospital’s biochemical indicators, the laboratory indices were revised. Because the adverse events related to new antineoplastic drugs such as ICIs (PD-1/PD-L1 monoclonal antibody) were not included, the triggers were supplemented with reference to relevant guidelines (4, 23), and 37 triggers were constructed.

Under the principles of informed consent and voluntary participation, we consulted the expert group, including one chief physician (oncology department), one attending physician (oncology department), three chief pharmacists, and one deputy chief pharmacist. All of them were senior practitioners with over 5 years of experience in their respective field. A total of six expert group members were consulted on the questionnaire, scored the importance of the initial triggers, include the ADEs in correspondence with the frequency of occurrence (0 for very rare to 5 for very common) (15) and the severity (0 for no harm to 5 for fatal) (22), and proposed suggestions for revision. Then, the triggers were revised according to the expert opinions, and 37 final versions were established, which include four modules: 22 laboratory indices, 10 clinical symptom, three antidotes, and two interventions. See **Table 1** for details.

**Table 1:** The triggers and PPV.

No.	Triggers	Associated ADE	Positive triggers (n)	ADEs (n)	PPV (%)
Laboratory index					
L1	Hb < 100 g L^−1^	Drug- associated anemia	116	107	92
L2	PLT < 100 * 10^9^ L^−1^	Drug-associated thrombocytopenia	86	74	86
L3	Neutrophils < 1.8 * 10^9^ L^−1^	Drug-associated neutropenia	143	131	92
L4	WBC < 4.0 * 10^9^ L^−1^	Drug-associated leukopenia	212	195	92
L5	ALT (or D-BIL) > 2 ULN or AST, ALP, and TBI increased simultaneously, at least one of them > 2ULN	Drug-associated hepatotoxicity	26	18	69
L6	Creatinine > 1.5 ULN or 1.5× baseline and/or GFR < 60 mL/min/1.73 m^2^	Drug-associated renal toxicity	5	4	80
L7	Blood pressure > 140/90 mmHg if previous values in the normal range	Drug-associated hypertension	105	3	2.9
L8	Fasting blood glucose > 8.9 mmol L^−1^ (not type 2 diabetes)	Drug-induced hyperglycemia	2	0	0.0
L9	Fasting blood glucose < 3. 9mmol L^−1^ (not type 2 diabetes)	Drug-induced hypoglycemia	2	0	0.0
L10	Blood potassium > 5.3 mmol L^−1^	Drug-induced hyperkalemia	2	0	0.0
L11	Blood potassium <3.5 mmol L^−1^	Drug-induced hypokalemia	58	1	1.7
L12	Blood calcium > 2.52mmol L^−1^	Drug-induced hypercalcemia	1	0	0.0
L13	Blood calcium < 2.11mmol L^−1^	Drug-induced hypocalcemia	130	0	0.0
L14	Blood uric acid (female > 360 μmol L^−1^ and male > 420 μmol L^−1^)	Drug-induced hyperuricemia	30	2	6.7
L15	Proteinuria, qualitative test of urine protein was positive or quantitative test for urine protein > 150 mg 24 h^−1^	Drug-induced proteinuria	11	3	27
L16	TnI > 0.034 ng mL^−1^ or CK-MB > 25U/L, CK > 200 U/L	Drug-induced myocardial damage	1	0	0.0
L17	BNP > 400 pg mL^−1^ or NT-proBNP> 2000 pg mL^−1^	Drug-induced cardiac failure	0	0	0.0
L18	TSH > 4.2 uIU mL^−1^, FT4 < 0.81 ng/dL	Drug-induced hypothyroidism	1	1	100
L19	TSH < 0.27 uIU mL^−1^, FT4 > 1.89 ng/dL	Drug-induced hyperthyroidism	1	1	100
L20	CT: pulmonary interstitial changes (ground glass nodules or patch nodules)	Drug-induced pulmonary injury	143	2	1.4
L21	TSH < 0.27 uIU mL^−1^, FT4 < 0.81 ng/dL	Drug-induced secondary hypothyroidism (hypophysitis)	0	0	0.0
L22	ACTH: 7 am to 10am < 7.2 pg mL^−1^; Cor, 6 am to 10 am < 6.02 µg dL^−1^	Drug-induced secondary adrenocortical hypofunction (pituitaritis)	0	0	0.0
Clinical symptom					
S1	Fever (temperature > 38.2°C)	Fever	25	11	44
S2	Oral mucositis	Oral mucositis	1	1	100
S3	Diarrhea (soft or liquid stools; >4 stools/day) and/or bellyache, hemoproctia, mucous stool, and/or fever	Diarrhea/colonitis	32	22	69
S4	Nausea and/or vomiting	Nausea/vomiting	70	64	91
S5	Constipation	Constipation	8	2	25
S6	Erythema, numbness, and desquamate of hands and feet	Hand-foot syndrome	3	3	100
S7	Limb distal paresthesia, pain, numbness, and hypoesthesia	Peripheral neuritis	17	16	94
S8	Rash (maculopapule pustular eruption)/itching	Drug-induced skin reactions	23	15	65
S9	Local skin pain, burning sensation, erythema, ulcer, and necrosis	Drug extravasation	0	0	0.0
S10	Skin surface nodular, patchy, and color bright red or dark red capillary hyperplasia	Reactive capillary hyperplasia	2	2	100
Antidote					
T1	Simultaneous use of glucocorticoids and antihistamines	Drug-induced allergy	180	2	1.1
T2	Anticoagulant drug	Drug-induced thrombosis	84	0	0.0
T3	Calcium leucovorin (except patients with colorectal and gastric cancer)	High dose methotrexate poisoning	3	2	67
Intervention					
M1	Unexpected emergency, rescue, and transfer to the ICU	ADEs required emergency, rescue, and transfer to the ICU	1	1	100
M2	Abrupt adjustment of antitumor therapy (drug withdrawal, dose reduction, or change chemotherapy regimen)	Treatment regimens need to be adjusted due to ADEs	32	32	100
**Subtotal**			1,556	715	46

PPV (number of ADEs identified with the trigger/number of positive triggers).

### 2.3 Retrospective records review

The medical records review panel consists of four members: two primary reviewers (pharmacist in charge) and two secondary reviewers (deputy chief pharmacist and chief pharmacist). Two primary reviewers independently reviewed the medical records, including the basic patient information, medical progress notes, nursing flow sheets, medication orders, and laboratory data. Each identified trigger was recorded for further chart analysis to determine whether an associated ADE had occurred. When an ADE is occurred, its respective category and severity are assigned. Secondary reviewers will assist the primary reviewers to complete the association evaluation between drugs and adverse events. When the ADEs are difficult to identify, the physician attests to the occurrence and severity of the ADEs and responds to the reviewer’s questions about the specific record. If there was a disagreement, then the final decision was made based on a consensus at the study group meetings. All researchers were trained to detecting ADEs using the trigger tool before starting work (7).

The sensitivity and specificity are used as the effective indicators for monitoring ADEs. The sensitivity is expressed as the positive trigger frequency (γ). The specificity was represented by the positive predictive value (number of ADEs identified with the trigger/number of positive triggers, PPV) and the detection rate of ADEs, which were as follows: (1) percent of admissions with ADE, (2) ADEs per 100 admissions, and (3) ADEs per 1,000 patient days.

The assessment of ADE causality was according to the Karch and Lasagna assessment method (24). The causality categories included: certain, probable, possible, suspicious (conditional), and impossible. Only cases with certain, probable, and possible levels were included in this study. The names of ADEs were standardized according to the medical dictionary for regulatory activities (MedDRA). The severity of ADEs was evaluated by CTCAE v5.0. Grade 1: Mild; asymptomatic or mild symptoms; clinical or diagnostic observations only; intervention not indicated. Grade 2: Moderate; minimal, local, or noninvasive intervention indicated; limiting age-appropriate instrumental activities of daily living (ADL). Grade 3: Severe or medically significant but not immediately life-threatening; hospitalization or prolongation of hospitalization indicated; disabling; limiting self-care ADL. Grade 4: Life-threatening consequences; urgent intervention indicated. Grade 5: Death related to ADE.

### 2.4 Statistical analysis

Microsoft Excel 2010 worksheet was used for data entry, and SPSS 25.0 software was used for statistical analysis. Descriptive statistics were calculated for patients and ADE characteristics. Categorical variables were summarized using frequency counts and percent, and continuous variables were presented as means with standard deviations (SD) and medians with ranges. Comparisons between groups were made using the t-test for continuous variables, and the χ2 test was used for categorical variables. A significance criterion of *P* < 0.05 was used in the analysis. The logistic regression model was used to analyze the risk factors of ADE and SAE. Any variable significant at a level of 0.05 after regression was reported as an independent risk factor for ADE or SAE.

## 3 Results

### 3.1 Patients characteristics

A total of 500 randomly selected patients were reviewed. Among these patients, 218 (43.6%) were women and the mean age was 63.11 ± 11.16 years (range, 29 to 86), and the age group of 45 to 69 years (63.2%) was the most. The average length of hospital stay was 6.53 ± 4.21 days (range, 2 to 28). The total length of hospitalization was 3,264 days. The most common type of tumor was digestive system tumor (260 cases, 52.0%), followed by gynecological malignant tumor (48 cases, 9.6%) and lung cancer (75 cases, 15.0%). The main tumor stage was III-IV (388 cases, 77.6%). Hypertension was the most common chronic comorbidities (251 cases, 50.2%), followed by diabetes mellitus (113 cases, 22.6%) and coronary heart disease (68 cases, 13.6%). The characteristics of patients are shown in **Table 2**.

**Table 2:** Patient characteristics.

Characteristics		Total (n = 500) (%)
Gender	Male	282 (56)
	Female	218 (44)
Age (years)	29–44	43 (8.6)
	45–69	316 (63)
	70–79	121 (24)
	≥80	20 (4.0)
Tumor type	Digestive system tumor	260 (52)
	Gynecological malignant tumor	48 (9.6)
	Lung cancer	75 (15)
	Lymphoma	38 (7.6)
	Breast cancer	33 (6.6)
	Urogenital system tumors	20 (4.0)
	Other tumor types	26 (5.2)
Tumor stage	I	17 (3.4)
	II	25 (5.0)
	III	87 (17)
	IV	301 (60)
	Small cell lung cancer localized stage	2 (0.4)
	Small cell lung cancer extensive stage	13 (2.6)
	Tumor not staging	55 (11)
Comorbidity	Hypertension	251 (50)
	Diabetes mellitus	113 (23)
	Coronary heart disease	68 (14)
	Cerebrovascular disease	49 (9.8)
	Hyperlipidemia	53 (11)
	Other diseases	225 (45)

Other tumor types included malignant pleural mesothelioma (nine cases, 1.8%), thymic carcinoma (five cases, 1.0%), head and neck squamous cell carcinoma (four cases, 0.8%), etc.

### 3.2 Triggers

Among the 37 triggers, 33 (89.2%) were positive, and 26 (70.3%) were associated with ADEs. A total of 459 records had at least one positive, and the rate of positive triggers (γ) was 91.8% (459/500), which indicated the sensitivity. There were a total of 1,556 positive triggering, and 715 ADEs (35 ADEs were identified by more than one trigger) were identified from 315 records. The specificity was as follows: the percent of admissions with ADE was (315/500, 63.0%), and the calculated rate of ADEs was 136.0 (95% CI, 124.12–147.88) per 100 admissions and 208.3 (95% CI, 201.20–215.46) per 1,000 patient days. The overall PPV was 46.0% (715/1,556). The PPV ranged from 0% to 100%, with a median of 44.0%, indicating that the specificity was good, as shown in **Table 1**.

### 3.3 ADE-related antineoplastic drugs

For the assessment of ADE causality, 180 (26.5%) cases were assessed as certain, 492 (72.4%) cases were assessed as probable, eight (1.2%) cases were assessed as possible.

For the ADE-related drugs, a total of 45 antineoplastic drugs involving seven categories. The top three categories were antimetabolic drugs (29.1%), plant sources and derivatives (27.1%), and metal platinum drugs (26.3%). The top three drugs were oxaliplatin (10.5%), fluorouracil (10.3%), and irinotecan (10.3%). The distribution of ADE-related drugs is shown in **Table 3**.

**Table 3:** Distribution of ADE-related antineoplastic drugs.

Categories of antineoplastic drugs	Suspected drugs	Counts (%)
Antimetabolic drugs	Fluorouracil (107), Gemcitabine (62), Capecitabine (41), Tegafur Gim eracil and Oteracil (31), Pemetrexed (25), Raltitrexed (15), Methotrexate (11), and Cytarabine (11)	303 (29)
Plant sources and derivatives	Irinotecan(107), Paclitaxel (albumin-binding type) (61), Paclitaxel (39), Etoposide (33), Vindesine (16), Paclitaxel liposome (13), Docetaxel (12), and Vincristine (2)	283 (27)
Metal platinum drugs	Oxaliplatin (109), Cisplatin (81), Carboplatin (62), Nedaplatin (16), and Lobaplatin (5)	273 (26)
Antitumor antibiotics	Epirubicin (36), Doxorubicin (14), and Pirarubicin (6)	56 (5.4)
Alkylating agent	Cyclophosphamide (37), Ifosfamide (11), and Bendamustine (7)	55 (5.3)
Molecular targeted drug	Bevacizumab (9), Regorafenib (7), Apatinib (6), Osimertinib (6), Cetuximab (5), Olaparib (5), Icotinib (4), Lenvatinib (3), Afatinib (2), Trastuzumab (2), Pyrrolitinib (1), Sorafenib (1), Niraparib (1), and Rituximab (1)	53 (5.1)
Immune checkpoint inhibitors	Tirelizumab (4), Carrilizumab (3), and Triplimab (2)	9 (0.9)
Total		1,032 (100)

Multiple suspected drugs may exist in the same adverse event, so the frequency of suspected drugs is greater than that of ADEs.

### 3.4 Characteristics of ADEs

For the categories of ADEs, 680 ADEs involve 12 categories. The most common category was the hematologic system (507/680, 74.6%), which mainly includes leukopenia, neutropenia, and anemia. The next category was gastrointestinal system (89/680, 13.1%), mainly as nausea and vomiting. For the severity grade of ADEs, 273 ADEs were grade 1 (273/680, 40.2%), 293 ADEs (293/680, 43.1%) were grade 2, 83 ADEs were grade 3 (83/680, 12.2%), 31 ADEs were grade 4 (31/680, 4.6%), and no grade 5 was found. The distribution of ADEs involved systems or organs and severity is shown in **Table 4**.

**Table 4:** Distribution of ADEs involved systems or organs and severity.

System/organ	Total counts (%)	Categories of ADEs	Counts	Classification of severity				
				1	2	3	4	5
Hematologic System	507 (75)	Anemia	107	–	87	20	–	–
		Leukopenia	195	91	73	23	8	–
		Neutropenia	131	33	61	23	14	–
		Thrombocytopenia	74	30	30	7	7	–
Gastrointestinal system	89 (13)	Nausea and/or vomiting	64	56	7	1	–	–
		Diarrhea/colonitis	22	9	11	2	–	–
		Oral mucositis	1	1	–	–	–	–
		Constipation	2	2	–	–	–	–
Skin and appendages	20 (2.9)	Hand-foot syndrome	3	1	2	–	–	–
		Rash	15	9	6	–	–	–
		Reactive capillary hyperplasia	2	2	–	–	–	–
Liver system	18 (2.7)	Drug-related hepatic injury	18	9	6	3	–	–
Peripheral nervous system	16 (2.4)	Peripheral neuritis	16	11	4	1	–	–
All over (the body)	11 (1.6)	Fever	11	11	–	–	–	–
Renal system	7 (1.0)	Drug-related renal injury	4	–	4	–	–	–
		Drug-induced proteinuria	3	1	1	1	–	–
Metabolism and nutrition	3 (0.4)	Drug-induced hypokalemia	1	1	–	–	–	–
		Drug-induced hyperuricemia	2	2	–	–	–	–
Cardiovascular system	3 (0.4)	Drug-induced hypertension	3	1	–	2	–	–
Endocrine system	2 (0.3)	Drug-induced hypothyroidism	1	1	–	–	–	–
		Drug-induced hyperthyroidism	1	1	–	–	–	–
Respiratory system	2 (0.3)	Drug-induced lung injury	2	1	1	–	–	–
Immune system	2 (0.3)	Relief drug allergy	2	–	–	–	2	–
Total	680 (100)		680	273	293	83	31	–

“-” indicates no associated ADEs.

### 3.5 Risk factors associated with the occurrence of ADE and SAE

Patients with ADE and those without ADE were evaluated, and the univariate statistical analysis showed that there was statistically significant difference in “age,” “the number of combined drugs,” “previous times of chemotherapy,” “previous history of ADR,” and “tumor stage” (*P* < 0.05). There was no statistically significant difference in “gender,” “length of hospital day,” “number of antitumor drugs,” “radiotherapy,” “combined with other diagnosis,” and “tumor type” (*P* > 0.05). A regression model was established. Multivariate logistic regression analysis showed that there was significant correlation between “the number of combined drugs (OR = 1.14; 95% CI, 1.07–1.22; *P* < 0.001),” “previous history of ADR (OR = 0.38; 95% CI, 0.23–0.60; *P* < 0.001),” and the occurrence of ADE (*P* < 0.05).

SAE is an adverse event that meets one or more of the following criteria: hospitalization (initial or prolonged), disability or permanent damage, congenital anomaly/birth defect, required intervention to prevent permanent impairment or damage (devices), life-threatening, death, and other serious important medical events (25). A total of 77 (77/500, 15.4%) patients were associated with SAE. The univariate statistical analysis showed that there was statistically significant difference between patients with and without SAE in “length of hospital day,” “the number of antitumor drugs,” and “the number of combined drugs” (*P* < 0.05). However, the results show that “previous history of ADR (χ2 = 3.75, *P*=0.053)” and “tumor type (χ2 = 9.46, *P* = 0.051)” may also be related to SAE occurrence in patients with tumor. Multivariate logistic regression analysis of the above factors showed that “length of hospital stay (OR = 0.40; 95% CI, 0.14–1.12; *P* < 0.05)” and “previous history of ADR (OR = 2.18; 95% CI, 1.07–4.45; *P* < 0.05)” were risk factors for SAE. The categories of ADEs were shown in **Table 4**.

## 4 Discussion

### 4.1 Trigger revision

In this study, 37 triggers were established on the basis of GTT and literature research and modified through experts’ investigation. For example, L6 “GFR < 60 mL/min or creatinine > 1.5 ULN”; GFR needs to be calculated and cannot be extracted directly from the medical record, and the item “creatinine > 1.5 times baseline” can be added according to CTCAE 5.0. Therefore, it was modified to “creatinine > 1.5 ULN or >1.5 times baseline and/or GFR <60 mL/min.” L8 “blood glucose > 8.9mmol L^−1^”; the patients with previous type 2 diabetes should be excluded and should be limited to fasting blood glucose; therefore, the item is modified to “fasting blood glucose > 8.9mmol L^−1^ (not type 2 diabetes),” and L9 is modified to “fasting blood glucose < 3.9mmol L^−1^ (not type 2 diabetes).” T3 “calcium leucorin” is designed to detect methotrexate poisoning. Because this drug is included in the treatment programs of colorectal cancer and gastric cancer, it is modified to “calcium leucorin (except patients with colorectal cancer and gastric cancer).” According to the expert opinion, the initial triggers are modified, and 37 final versions are established. An antitumor drug adverse event screening tool suitable for our medical institution was constructed.

The application of the triggers in ADEs monitoring of patients with cancer in our hospital, and the results showed that it was effective in monitoring antitumor drug adverse events. Although the positive trigger frequency has been higher than many domestic and foreign studies (10, 12), there are still four (4/37, 10.8%) whose positive trigger frequency is 0, and they are L17, L21, L22, and S9, respectively; L17 shows heart failure adverse events caused by antitumor drugs, and anthracyclines and trastuzumab have the highest risk of cardiotoxicity (26). In the collected cases, most of the patients who received anthracyclines were given dexrezzo to prevent cardiotoxicity after reaching a certain cumulative dose. No cardiotoxicity caused by trastuzumab was identified, which may be related to the insufficient sample size. More records should be screened to further confirm the application value of the trigger. L21 and L22 are mainly used for the adverse events of pituitaritis caused by the new antitumor drug PD-1/PD-L1 preparation, which is a common irAE in patients with cancer treated with PD-1/PD-L1 (27). The failure to be identified may be related to the insufficient application of PD-1/PD-L1 preparation in our hospital. Subsequent use of these drugs will gradually increase, so it is recommended to retain these triggers. The clinical manifestations of S9, such as local pain and erythema, are related to extravasation of chemotherapy drugs, which is easy to be confused with the clinical manifestations of other adverse reactions and has low specificity. It is suggested to be modified.

The overall PPV (46.0%) value of this study is between domestic and foreign similar studies (13, 15, 18). However, some of the PPV are low, for example, L7 (blood pressure > 140/90 mmHg) positive trigger frequency is high, and the PPV value is only 2.9%, which may be due to the high proportion of patients with basic hypertension diseases (251/500, 50.2%), which are mostly caused by primary disease rather than antitumor agents. The positive trigger frequency of T1 is high, and the PPV value is only 1.1%, probably because, in order to prevent the occurrence of severe allergic reactions, some antitumor drugs (such as paclitaxel) need to be pretreated with dexamethasone and antihistamines. T2 was used to detect thrombotic adverse events caused by antitumor drugs, although the positive trigger frequencies were 84, but the PPV value was 0, the reason is that patients with tumor were prone to hypercoagulability and cause tumor-associated thrombosis. However, anti-angiogenic drugs (bevacizumab, etc.) were easy to cause thromboembolic adverse events (28); therefore, there is a recommendation to retain T2. The positive trigger frequencies of L8, L9, L10, and L12 was low; the PPV value was 0, the low specificity suggested that the above triggers were less involved in antitumor drugs, and it was advised to delete later. The positive trigger frequencies of L11 and L13 were higher, whereas the PPV was only 1.7% and 0, which may be due to the large consumption of patients with tumor and poor nutritional status, leading to more patients with hypokalemia and hypocalcium, rather than the correlation with antitumor drugs. Thus, it was also advised to delete later. L16 was positively triggered in only one case, but antitumor drugs, especially PD-1/PD-L1 preparations, which could cause myocardial injury and the mortality is high (29, 30), so it is recommended to retain it. The study results showed that the PPV of L18, L19, S2, S6, and S10 was 100%, but, due to the low positive trigger frequency (≤3), the calculated PPV value may have a large deviation; therefore, more medical records should be screened for specificity analysis and verification in the later period.

### 4.2 Characteristics of ADE occurrence

In this study, the rates of ADEs (63.0%) and SAE (15.4%) were detected. The hematologic system was most common, mainly manifested as leukopenia, neutropenia, and anemia, followed by the gastrointestinal system. In addition, the severity of ADEs was mainly mild or moderate (83.2%). The results are similar to the other study (13). However, in a French study of 1,000 patients with solid tumors undergoing chemotherapy, the incidence rate of SAE was 44.5%, of which 27.9% were hematological disorders (31). A study from Thailand conducted a prospective observational study on 151 elderly patients with tumor (aged ≥ 70 years) undergoing chemotherapy (32), and the results showed that the incidence rate of ADEs was 83.4% and that of SAE was 42.4%, of which 27.1% were severe hematological toxicity, 37.7% were neutropenia, and 22.5% anemia. It is different with the results of our study, which may be related to the different basic characteristics and clinical environment of the enrolled patients. For example, the patients of the study from Thailand were older, and the triggers such as fatigue, infection, and paronychia were not included in our study. Furthermore the threshold of laboratory indicators was low, such as abnormal transaminase and creatinine clearance, which could be identified as ADEs. In this study, most antitumor drugs with a high risk of ADE were given corresponding prophylactic drugs, and the threshold of laboratory indicators is higher, so the incidence of ADEs detected in this study was lower. The incidence of ADE is related to multiple factors and depends on the tumor type, clinical background, and drugs administered. The drugs of this study not only include chemotherapy drugs but also include molecular targeting drugs and ICIs, so the incidence of ADE and SAE differs from other studies. The studies (11, 21) have shown that functional status score, age (>72 years old), moderate to severe complications, gastrointestinal or genitourinary tumors, low hemoglobin, long-term hospitalization (>7 days), impaired renal function, and multiple drug use (>8 types/day) are the risk factors for ADEs in chemotherapy patients. Physical condition score is risk factor for SAE. In this study, multivariate logistic regression analysis showed that the number of combined medications and the previous history of ADR were the independent risk factors for ADE, whereas the length of stay and the previous history of ADR were the independent risk factors for SAE.

### 4.3 ADE-related drugs

The top three categories of the ADE-related drugs were antimetabolic drugs (29.1%), plant sources and derivatives (27.1%), and metal platinum drugs (26.3%). The top three drugs were oxaliplatin (10.5%), fluorouracil (10.3%), and irinotecan (10.3%). In the extracted medical records, digestive system tumors were the most common tumor types. Oxaliplatin, fluorouracil, and irinotikan are widely used antitumor drugs in digestive system tumors, which accounts for a higher proportion of ADE-related drugs. A study analyzed 15,183 ADR reports of antitumor drugs (33), and the results showed that the top three categories of antitumor drugs were platinum metals, plant sources and derivatives, and antimetabolic drugs, which were similar to the results of this study.

## 5 Conclusions

This was one of the few studies that have looked into detect ADEs in patients with tumor through establishing a trigger tool based on GTT in China. The study established 37 triggers to detected ADEs by retrospective review. The methodology established could be used to monitor antineoplastic drugs adverse events in patients with tumor effectively. Our results showed that the sensitivity was better and the specificity was good. The number of combined medications and the previous history of ADR were the independent risk factors for ADE, whereas the length of stay and the previous history of ADR were the independent risk factors for SAE.

There are several limitations in the present study. For example, ADE detection was based solely on a retrospective review of the medical records. The results were dependent on the quality of the documentation, which varied across different departments and doctors, and some mild and rare ADEs were likely to be overlooked by the clinician and not recorded in the medical records. In addition, this study only enrolled medical records of hospitalized patients, whereas some oral antitumor drugs (e.g., small-molecule targeted drugs) may be taken in the outpatient clinic and therefore difficult to monitor. In addition, the limitation of the study is that it was conducted in only one hospital. Despite the limitations of this study, the established trigger tool may detect ADEs effectively but still needs to be optimized. This study may provide some references for further research in order to improve the rationality and safety of antineoplastic medications. In the future, the trigger tool could be incorporated into routine screening systems to provide real-time identification of ADEs, thereby enabling initiation of timely clinical interventions.

## Data availability statement

The original contributions presented in the study are included in the article/supplementary material. Further inquiries can be directed to the corresponding author.

## Ethics statement

Ethics approval was obtained from the respective ethics committees of Beijing Luhe Hospital, Capital Medical University (NO.2021-LHKY-091-01). The studies were conducted in accordance with the local legislation and institutional requirements. Written informed consent for participation was not required from the participants or the participants’ legal guardians/next of kin in accordance with the national legislation and institutional requirements.

## Author contributions

All the authors participated in the study. YL, XL, and HC contributed to the design of the study. YL, BX, JC, and WS participated in the literature search and the trigger revision and conducted the experts’ investigation. YL, XL, BX, FL, and HC participated in the review of retrospective records and the data analysis. YL and HC participated in the writing and revising of the manuscript. All authors contributed to the article and approved the submitted version.
